# Progressive Gender Differences of Structural Brain Networks in Healthy Adults: A Longitudinal, Diffusion Tensor Imaging Study

**DOI:** 10.1371/journal.pone.0118857

**Published:** 2015-03-05

**Authors:** Yu Sun, Renick Lee, Yu Chen, Simon Collinson, Nitish Thakor, Anastasios Bezerianos, Kang Sim

**Affiliations:** 1 Singapore Institute for Neurotechnology (SINAPSE), Centre for Life Sciences, National University of Singapore, Singapore, Singapore; 2 Department of Bioengineering, National University of Singapore, Singapore, Singapore; 3 Department of Engineering Science, National University of Singapore, Singapore, Singapore; 4 Department of Psychology, National University of Singapore, Singapore, Singapore; 5 Department of General Psychiatry, Institute of Mental Health (IMH), Singapore, Singapore; 6 Department of Research, Institute of Mental Health (IMH), Singapore, Singapore; Beijing Normal University, CHINA

## Abstract

Sexual dimorphism in the brain maturation during childhood and adolescence has been repeatedly documented, which may underlie the differences in behaviors and cognitive performance. However, our understanding of how gender modulates the development of structural connectome in healthy adults is still not entirely clear. Here we utilized graph theoretical analysis of longitudinal diffusion tensor imaging data over a five-year period to investigate the progressive gender differences of brain network topology. The brain networks of both genders showed prominent economical “small-world” architecture (high local clustering and short paths between nodes). Additional analysis revealed a more economical “small-world” architecture in females as well as a greater global efficiency in males regardless of scan time point. At the regional level, both increased and decreased efficiency were found across the cerebral cortex for both males and females, indicating a compensation mechanism of cortical network reorganization over time. Furthermore, we found that weighted clustering coefficient exhibited significant gender-time interactions, implying different development trends between males and females. Moreover, several specific brain regions (e.g., insula, superior temporal gyrus, cuneus, putamen, and parahippocampal gyrus) exhibited different development trajectories between males and females. Our findings further prove the presence of sexual dimorphism in brain structures that may underlie gender differences in behavioral and cognitive functioning. The sex-specific progress trajectories in brain connectome revealed in this work provide an important foundation to delineate the gender related pathophysiological mechanisms in various neuropsychiatric disorders, which may potentially guide the development of sex-specific treatments for these devastating brain disorders.

## Introduction

Accumulated neuroimaging evidence has shown the presence of sexual dimorphism in brain structures that could underlie sex differences in behavior and cognition [1,2]. For instance, male brains are larger in size with comparatively higher white matter percentage, while females exhibit greater gray matter (GM) percentage after correcting for intracranial volume effect [3]. Sex differences have also been reported in specific brain structures, including corpus callosum, frontal forceps, and hippocampus [4]. Furthermore, the normal brain undergoes considerable morphological changes with age, which is thought to account for the age related decline in cognitive domains such as working memory, speed of information processing, and long-term memory [5]. Convergent evidence has emerged to show different developmental trajectories of the brain in males and females [6,7]. Nonetheless, previous longitudinal studies mainly examined brain development during childhood or adolescence [8–10] or focused on GM changes over time [11,12]. In healthy adults, gender differences in brain structures (especially involving white matter (WM)) have been largely derived from cross-sectional work [13,14] and there is a need for longitudinal investigation in order to provide a more comprehensive understanding of sex-specific architectural change that occurs throughout life [15].

A recent conceptualization suggests that the brain can be considered as a large-scale network of interconnected nodes within the human connectome [16]. Advances in diffusion tensor imaging (DTI) technique and graph theory methods have enabled quantitative mapping of brain anatomical network at different scales of investigation [17,18], and it has recently been applied to the study of gender difference [19,20]. Specifically, structural networks of both genders have been shown to exhibit an economical ‘small-world’ character (defined as manifesting high local clustering coefficient and short path length between nodes) as well as highly connected individual network hubs [19,20]. One recent study on a large sample of youths revealed higher within-hemispheric connectivity in males whereas between-hemispheric connectivity was found predominantly in females [21]. It is noteworthy that to date studies are exclusively cross-sectional. A longitudinal design could therefore extend this work and lead to greater elucidation of the developmental topological differences in brain structure between adult males and females.

To the best of our knowledge, this is the first study employing graph theoretical analysis to investigate longitudinal gender effects on structural brain networks. We calculated several network measures to assess the small-worldness property, network efficiency, and relative nodal importance within the networks and investigated their associations with sex and across time. Based on prior research on sexual dimorphism and normal development of brain connectome, we hypothesized: (*i*) that brain networks would exhibit sex related differences, (*ii*) that brain networks would become less efficient over time, and (*iii*) that there was progressive sexual dimorphism in brain topological organization and regional characteristics.

## Methods and Materials

### Participants

All participants gave written informed consent to the research protocol, which was approved by the Institutional Review Board of the Institute of Mental Health, Singapore as well as that of National Neuroscience Institute, Singapore. Ethics review criteria conformed to the Declaration of Helsinki. We separated the subjects into baseline and longitudinal groups, the purposes of which were (*i*) to verify the sexual dimorphism of structural brain networks in a cross-sectional manner and (*ii*) to investigate the progressive gender differences of brain connectome over time.

**Baseline:** Participants for this study were seventy-one healthy subjects (43 males: 30.0 ± 7.5 years old, range 22–53, right-hand/left-hand: 40/3; and 28 females: 33.7 ± 12.1 years old, range 21–59, right-hand/left-hand: 26/2), recruited from the local community by advertisements. At inclusion, subjects would be excluded if they met any of the following clinical characteristics: (a) history of any major medical/neurological illness, including brain trauma, epilepsy, or cerebral vascular accident; (b) substance abuse or psychotropic medication use; (c) Axis I psychiatric disorder according to the Structural Clinical Interview for Diagnostic and Statistical Manual of Mental Disorder, Fourth Edition (DSM-IV) Non-Patient version (SCID-NP); (d) have a first-degree relative with any mental illness.

**Longitudinal:** among the 71 subjects at inclusion, twenty-eight subjects (13 males: 36.0 ± 6.6 years old, range 29–53, right-hand/left-hand: 11/2; and 15 females: 38.8 ± 11.5 years old, range 26–61, right-hand/left-hand: 14/1) completed the longitudinal study and underwent rescanning after an average interval of 5 years (mean ± S.D. = 5.1 ± 1.0 years).

For both of the subject groups, there were no statistical differences (all p > 0.05) between the male and female participants with respect to age at scan, handedness, education level, and the time interval between scans (for the longitudinal group).

### Image acquisition

Structural MR images of the brain corresponding to precise guidelines to ensure consistent high signal-to-noise ratio were recorded using a 3-Tesla whole body scanner (Philips Achieva, Philips Medical System, Eindhoven, The Netherlands) with a SENSE head coil in National Neuroscience Institute, Singapore. A T1-weighted Magnetization Prepared Rapid Gradient Recalled sequence (repetition time [TR] = 7.2 s; echo time [TE] = 3.3 ms; flip angle = 8°) was utilized to obtain high-resolution T1-weighted MRI volume images (each volume contains 180 axial slices of 0.9 mm thickness, gapless axial slices; field of view = 230×230 mm; acquisition matrix = 256×256; in-plane resolution = 0.9×0.9 mm) in the direction of the anterior-posterior commissures. A single-shot echo-planar sequence (TR = 3275 ms; TE = 56 ms; flip angle = 90°; b-factor = 800 s/mm^2^) from 15 separate non-parallel directions was utilized to obtain diffusion encoded images (one baseline image without diffusion weighting, b0 = 0 s/mm^2^; each containing 42 slices, 3.0 mm, gapless axial slices; field of view = 230×230 mm; acquisition matrix = 112×109, reconstructed to 256×256). Three volumes (3 excitations) were procured to improve the signal-to-noise ratios. The structural and diffusion tensor images were obtained in order within a single scan time without altering position. Head motion was minimized using restraining foam pads provided by the manufacturer. The settings were maintained for both baseline and follow-up studies.

### Data preprocessing and network construction

Diffusion image preprocessing and structural brain network construction was conducted using FSL [22], diffusion toolkit [23] and a pipeline tool, PANDA [24]. Briefly, for each subject, DTI images were first corrected for motion and eddy current distortions using affine transformation to the b0 image. After this process, six elements of the diffusion tensor were estimated, from which fractional anisotropy (FA) was calculated. Whole-brain fiber tractography was subsequently performed using fiber assignment by continuous tracking (FACT) algorithm [25]. This algorithm computes fiber trajectories starting from the deep WM regions and terminating at a voxel with a turning angle > 45° or reached a voxel with an FA of < 0.15.

The method used to estimate the anatomical connections was similar to the procedure adopted by Gong et al. [26]. Nodes are the fundamental building blocks constituting brain network construction. In this study, we mapped 90 cortical and subcortical regions (45 in each hemisphere) to the respective nodes according to the automated anatomical labeling (AAL) parcellation scheme [27]. The names and corresponding abbreviations of the cortical regions were listed in Table 1. For each subject, the parcellation process was conducted in the native DTI space. Specifically, a linear transformation was applied locally within each subject’s DTI image correlated with T1-weighted image to coregister them to the b0 image in the DTI space followed by applying a nonlinear transformation to map to the ICBM152 T1 template (Montreal Neurological Institute). The subject-specific AAL mask was then weaved from the MNI space to the DTI native space with the corresponding inverse transformation such that separate labeling values were maintained via nearest neighbor interpolation [24,26].

**Table 1:** Abbreviations of cortical and subcortical regions defined in Automated Anatomical Labeling (AAL) template image in standard stereotaxic space.

Region name	Abbreviation	Region name	Abbreviation
Precentral gyrus	PreCG	Lingual gyrus	LING
Superior frontal gyrus (dorsal)	SFGdor	Superior occipital gyrus	SOG
Orbitofrontal cortex (superior)	ORBsup	Middle occipital gyrus	MOG
Middle frontal gyrus	MFG	Inferior occipital gyrus	IOG
Orbitofrontal gyrus (middle)	ORBmid	Fusiform gyrus	FFG
Inferior frontal gyrus (opercula)	IFGoperc	Postcentral gyrus	PoCG
Inferior frontal gyrus (triangular)	IFGtriang	Superior parietal gyrus	SPG
Orbitofrontal gyrus (inferior)	ORBinf	Inferior parietal lobule	IPL
Rolandic operculum	ROL	Supramarginal gyrus	SMG
Supplementary motor area	SMA	Angular gyrus	ANG
Olfactory	OLF	Precuneus	PCUN
Superior frontal gyrus (medial)	SFGmed	Paracentral lobule	PCL
Orbitofrontal gyrus (medial)	ORBmed	Caudate nucleus	CAU
Gyrus rectus	REC	Putamen	PUT
Insula	INS	Pallidium	PAL
Anterior cingulate gyri	ACG	Thalamus	THA
Middle cingulate gyri	MCG	Heschl gyrus	HES
Posterior cingulate gyrus	PCG	Superior temporal gyrus	STG
Hippocampus	HIP	Temporal pole (superior)	TPOsup
Parahippocampal gyrus	PHG	Middle temporal gyrus	MTG
Amygdala	AMYG	Temporal pole (middle)	TPOmid
Calcarine cortex	CAL	Inferior temporal gyrus	ITG
Cuneus	CUN		

**Fig 1:**
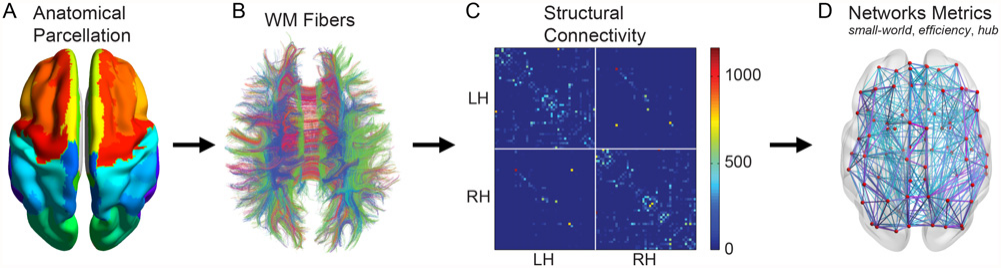
Schematic overview of the formation of the individual structural brain network. (A) Individual T1 images were used for parcellation of the cortex into 90 distinct brain regions through Automatic Anatomical Labeling (AAL) template, which form the nodes of the structural brain networks. (B) White matter fiber reconstruction in the whole cerebral cortex was performed using the fiber assignment by continuous tracking (FACT) algorithm. (C) The weighted structural brain network of each subject was created by computing the fiber numbers that connected each pair of brain regions. (D) The topological organization of the resulting individual structural brain networks was examined using graph theoretical measures.

The number of the connected fibers between two regions was defined as the weight of the network edges [28,29], which was estimated based on the whole brain tractography to represent the anatomical networks among all cortical regions. As a result, we constructed the weighted structural brain network for each participant, which was represented by a symmetric 90×90 matrix (Fig. 1). To eliminate false-positive brain connections due to possible noise effects on the whole brain tractography, a predefined threshold was chosen: if the fiber number (FN) between the two regions was larger than 3; these two regions were considered connected [29]. All of the resulting networks were fully connected with no isolated nodes. Then the obtained structural brain networks were analyzed using Brain Connectivity Toolbox [17]. Here, we mainly focused on the results estimated from the fiber number based structural connectivity networks. We also validated our observations on the FA-based structural connectivity matrices (see S1 Text).

### Network analysis

**Global network metrics.** Six network metrics (including strength, *S*_*p*_, weighted clustering coefficient, *C*_*p*_, weighted characteristic path length, *L*_*p*_, small-worldness, *σ*, global efficiency, *E*_*global*_, and local efficiency, *E*_*local*_), were adopted to characterize the global topological organization of structural brain networks.

For a given network *G* with *N* nodes, the strength is defined as follows:

$$S_{p}=\frac{1}{N}\sum_{i\in N}S_{p}\left(i\right),$$ (1)

where *S*_*p*_(*i*) is the sum of the edge weights linking to node *i*. Given that male brains are larger in size with comparatively higher white matter percentage [3], in order to investigate the progressive gender differences in the topological architectures beyond the simple gender-related differences in WM connectivity strength, a normalization approach was applied here to control the influences of the total fiber number differences [28,30]. Specifically, the structural brain network of each participant was normalized by its *S*_*p*_ prior to the estimation of the graph theoretical metrics.

The weighted clustering coefficient *C*_*p*_ is defined as [31]:

$$C_{p}=\frac{1}{N}\sum_{i\in N}\frac{2\sum_{j,k}{\left(w_{ij}w_{jk}w_{ki}\right)}^{1/3}}{\left(k_{i}\left(k_{i}-1\right)\right)},$$ (2)

where *k*_*i*_ is the number of edges connecting node *i*, and *w*_*ij*_ is the edge weight, which is scaled by the mean of all weights to control each participant’s cost at the same level [20,28]. A network with high *C*_*p*_ value has tightly connected local clusters and hence the loss of an individual node has an impact on the structure of the network.

Path length of an edge conceptualized to weight graphs is defined as the reciprocal of the edge weight. The weighted characteristic path length *L*_*p*_ of a network is defined as:

$$L_{p}=\frac{1}{N\left(N-1\right)}\sum_{i\in N}\sum_{i\neq j\in N}\min\left\{L_{ij}\right\},$$ (3)

where min{*L*_*ij*_} is the shortest path length between node *i* and node *j*. *L*_*p*_ reflects the mean distance or overall routing efficiency between any given pair of nodes [31].

The “small-world” network topology was introduced by Watts and Strogatz in their influential work of graph theoretical analysis of neural networks of the worm C. elegans [31]. The small-worldness metric of a network could be estimated as:

$$\sigma =\frac{\gamma }{\lambda },$$ (4)

where *γ* = *C*_*p*_/*C*_*p*_^*rand*^ is the normalized clustering coefficient and the *λ* = *L*_*p*_/*L*_*p*_^*rand*^ is the normalized characteristic path length, in which *C*_*p*_^*rand*^ and *L*_*p*_^*rand*^ denote the average clustering coefficient and the average characteristic path length of an ensemble of 100 surrogate random networks [31]. These random networks were derived from the original brain network by randomly rewiring the edges between nodes while preserving the degree distribution and connectedness [32]. We retained the weight of each edge during the random rewiring procedure. A real network is considered small-world if it meets the following criteria: *γ* ≫ 1 and *λ* ≈ 1 [31], or *σ* = *γ* /*λ* >1 [33].

The global efficiency *E*_*global*_ of *G* is defined by the inverse of the harmonic mean of the shortest path length between each pair of regions [34,35]:

$$E_{global}=\frac{1}{N\left(N-1\right)}\sum_{i\neq j\in N}\frac{1}{\min\left\{L_{ij}\right\}}.$$ (5)

*E*_*global*_ quantifies the efficiency of the parallel information transfer in the network. In contrast, the local efficiency of a node *i* was calculated as the global efficiency *E*_*global*_(*i*) of the neighborhood sub-network of this node, indicating how well the information can be communicated among the first neighbors of the node *i* when it is removed. The local efficiency across all nodes was averaged to estimate the local efficiency of the network:

$$E_{local}=\frac{1}{N}\sum_{i\in N}E_{global}\left(i\right).$$ (6) *E*_*local*_

represents how much the complex network is fault tolerant.

**Regional nodal characteristics.** Nodal betweenness centrality was adopted in this study to examine the regional characteristics of the structural brain networks. The betweenness centrality *bc*_*i*_ of a node *i* is defined as [36]:

$$bc_{i}=\sum_{j\neq i\neq k\in G}\frac{\delta_{jk}\left(i\right)}{\delta_{jk}},$$ (7)

where *δ*_*jk*_ is the number of shortest paths from node *j* to node *k*, and *δ*_*jk*_(*i*) is the number of shortest paths between node *j* and node *k* that pass through node *i*. We calculated the normalized betweenness as *BC*_*i*_ = *bc*_*i*_/〈*bd*_*i*_〉, where 〈*bd*_*i*_〉 was the average betweenness of all nodes. Hence, the *BC*_*i*_ captures the influence of a node over information flow between other nodes in the network. Regions with a high betweenness centrality indicate high interconnectivity with other regions in the network. Region *i* was identified as hub if *BC*_*i*_ was at least one standard deviation greater than the average of the metrics over the network (*BC*_*i*_ > mean + S.D.).

### Statistical analysis

Statistical analyses were performed using SPSS (version 17.0, IBM, Armonk, New York). Data were first checked for normality and transformed when necessary to meet the assumption of normal distribution. Outlier detection was performed through the Grubbs’ test and further validated through the boxplot. Significant outlier would be removed for the statistical analysis. To assess the significance of group differences in network topology at *baseline* group (total 71 participants, male/female = 43/28), a univariate analysis of covariance (ANCOVA) was performed separately on the following dependent variables: six global network metrics and regional nodal metrics for each region. Subject’s age at scan, handedness, and brain size were set as covariates. To determine whether there were significant longitudinal effects on the network properties, a two-way general linear model (GLM) was generated, comprising of gender as a between-subject factor, scan time point as a within-subject factor, and the gender by time as interaction. Age at scan, handedness, and brain size were set as covariates. This model was fitted to the network properties obtained in *longitudinal* group. If any main effect was found to be significant, further post-hoc *t*-tests were performed (paired *t*-test for longitudinal time effect and two-sample two-tailed *t*-test for gender effect). The threshold value for establishing significance was set at p < 0.05. Because of the exploratory nature of the current study, corrections for multiple comparisons of nodal characteristics were not performed.

## Results

### Gender effect on the global properties at baseline

We first studied the gender differences in the network topology at baseline. We found that the brain networks of both male and female exhibited typical small-world properties as expressed by a much higher local clustering than random networks while a comparable short characteristic path length of the random networks (see S1 Fig.). Significant gender effect was revealed in the strength, *S*_*p*_, (*male>female*; F_1, 65_ = 8.705, p = 0.004), indicating higher overall WM fiber numbers in male subjects. After controlling the effects of different numbers of total fiber on the topological differences between male and female, significant sex effects were found in small-worldness, *σ*, (*male<female*; F_1, 65_ = 6.343, p = 0.014), and global efficiency, *E*_*global*_, (*male>female*; F_1, 65_ = 4.334, p = 0.041). The other global network metrics (*C*_*p*_, *L*_*p*_, and *E*_*local*_) failed to pass the significance level. Notably, these statistical results were obtained after factoring out the effect of brain size. Therefore, the obtained significant results represented the salient gender effect on the topological organization of structural brain network independent of their difference in brain size. Taken together, these results demonstrated that males tended to be more globally efficient for information transfer (*E*_*global*_: *male>female*) while females tended to be more economical in small-world architecture (*σ*: *male<female*).

### Gender effect on the regional properties at baseline

**Fig 2:**
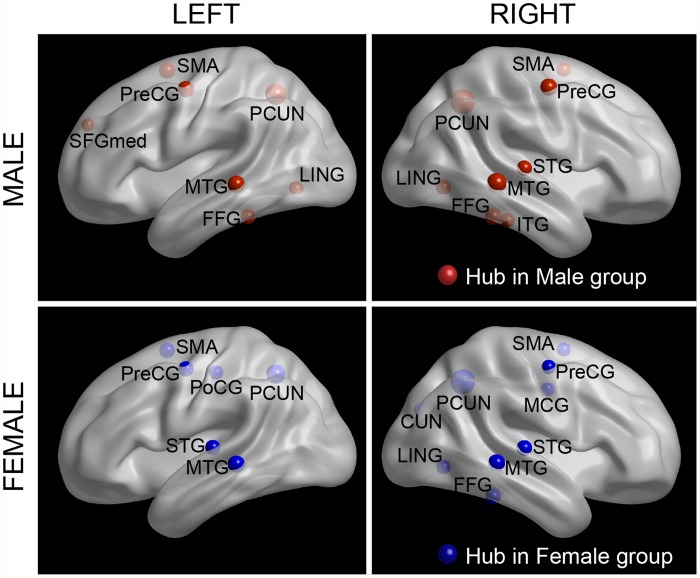
The global network hubs with high betweenness centrality in male subjects and female subjects. Regions with betweenness centrality one S.D. greater than the average over the network were identified as hubs. The hub nodes are shown in red (male) and blue (female) with node size representing their normalized nodal betweenness centrality. The regions were overlaid on the brain surface at the Medium view. The nodal regions are located according to their centroid stereotaxic coordinates. The figures were visualized with BrainNet Viewer software (http://www.nitrc.org/projects/bnv/).

**Fig 3:**
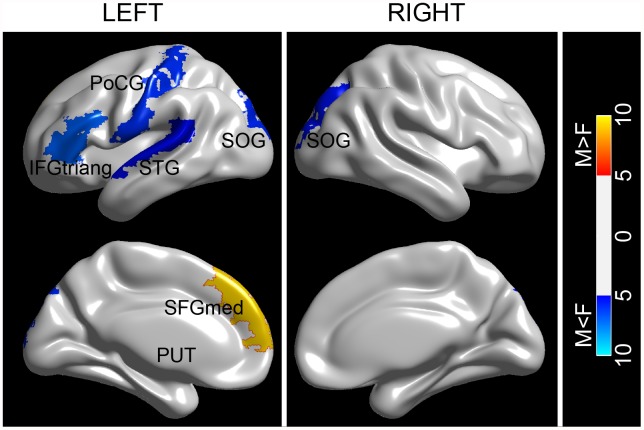
The spatial distribution of cortical regions showing significant gender effect. The color bar represents F values of group comparison. L and R represent left and right hemisphere, respectively. For the abbreviations of the cortical regions, see Table 1. The figures were visualized with BrainNet Viewer software (http://www.nitrc.org/projects/bnv/).

In this study, we identified hubs according to the normalized nodal betweenness centrality—a measurement that captures the influence of a node on information flow between other nodes in the network. As shown in Fig. 2, fifteen regions were designated as the hubs because of their large values of *BC*_*i*_ in men (Fig. 2(A)) and women (Fig. 2(B)). Most of the hub regions (11 out of 15) were overlapped between males and females, among which four cortical regions (the supplementary motor area [SMA], the precuneus [PCUN], the precentral gyrus [PreCG], and the middle temporal gyrus [MTG]) appeared as hubs in a bilaterally symmetric fashion. In addition, four brain regions (the left superior frontal gyrus [SFGmed.L], the left lingual gyrus [LING.L], the left fusiform gyrus [FFG.L], and the right inferior temporal gyrus [ITG.R]) were considered as hubs only for male subjects; whereas four different brain regions (the left postcentral gyrus [PoCG.L], the left superior temporal gyrus [STG.L], the right middle cingulate gyri [MCG.R], and the right cuneus [CUN.R]) were identified as hubs only in the female group. Among the hub regions identified for both groups, fifteen regions were association cortices, suggesting their critical roles in receiving convergent inputs from multiple cortical regions [37]. Despite the high degree of overlapping of hub regions between male and female subjects, further statistical analysis revealed significant gender effects on nodal characteristics of seven cortical regions (Fig. 3). Most of these regions (five out of seven) showed significant *female>male* effect; including (the left inferior frontal gyrus, the triangular part [IFGtriang.L], the PoCG.L, the STG.L, and bilateral superior occipital gyrus [SOG]). Regions with significant *female<male* effect were the SFGmed.L and the left putamen (PUT.L).

### Longitudinal gender effect on the global properties

**Fig 4:**
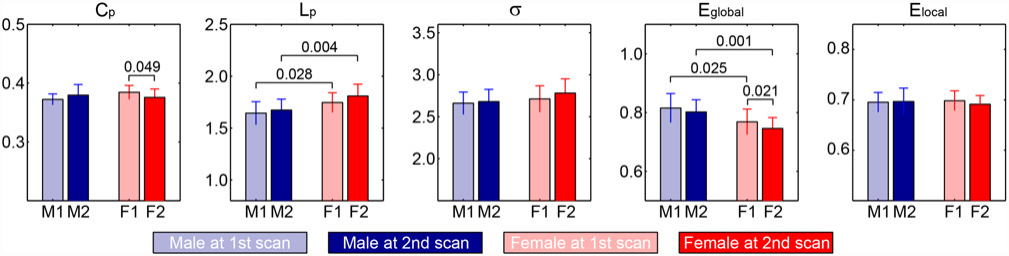
Post-hoc statistical analysis of global network metrics. Bars represent mean ± standard deviations. Each horizontal line and associated number represent the p-value of a *t*-test (paired *t*-test for longitudinal time effect, and two-sample *t*-test for gender effect). Males and females are marked separately: M1 = males at 1^st^ scan (light blue), M2 = males at 2^nd^ scan (blue), F1 = females at 1^st^ scan (light red), and F2 = females at 2^nd^ scan (red).

Prior to the statistical analysis, two subjects (one male and one female) were identified as outliers on the global properties. Therefore, longitudinal changes in global network topology were assessed in the remaining twenty-six subjects (male/female = 12/14). Similar to the baseline results, significant gender main effect was revealed in strength, *S*_*p*_ (*male>female*, F_1, 20_ = 5.657, p = 0.027), *L*_*p*_ (*male<female*, F_1, 20_ = 14.431, p = 0.001), small-worldness, *σ*, (F_1, 20_ = 8.605, p = 0.008) and *E*_*global*_ (*male>female*, F_1, 20_ = 13.052, p = 0.002) (Table 2). For longitudinal time effect, we observed ongoing decreases in *S*_*p*_ (F_1, 24_ = 8.110, p = 0.009) as well as decrease trends in *L*_*p*_ (F_1, 24_ = 3.378, p = 0.079) and *E*_*global*_ (F_1, 24_ = 3.652, p = 0.068), whereas the rest of the global network metrics did not show any effect over time. Results of post-hoc *t*-tests are shown in Fig. 4. Here only those global network metrics that exhibit significant main effect are presented. When examining the with-in subject changes, we found that males and females had a remarkably similar evolution pattern for most global network metrics. For instance, a progressive impairment in global efficiency of parallel information transfer with preserved local information integration and small-worldness properties was revealed in both groups. Interestingly, there was a significant gender-time interaction on *C*_*p*_ (F_1, 24_ = 4.333, p = 0.048). Further *t*-test analysis indicated that this interaction resulted from an insignificant increase of *C*_*p*_ in males (1^st^ *vs*. 2^nd^, t_11_ = -1.868, p = 0.089) and a significant decrease in females (1^st^ *vs*. 2^nd^, t_13_ = 2.174, p = 0.049) overtime.

**Table 2:** Comparison of network metrics with longitudinal scans between male (n = 12) and female (n = 14).

	Gender	Time	Gender × Time
Metrics	F_1, 20_ (p-value)	F_1, 24_ (p-value)	F_1, 24_ (p-value)
*S*_*p*_	**5.657 (0.027)** ^**F<M**^	**8.110 (0.009)** ^**1st>2nd**^	0.033 (0.857)
*C*_*p*_	0.100 (0.756)	0.151 (0.701)	**4.333 (0.048)**
*L*_*p*_	**14.431 (0.001)** ^**F>M**^	*3*.*378* (*0*.*079*)^1st<2nd^	0.439 (0.514)
*σ*	**8.605 (0.008)** ^**F>M**^	1.912 (0.180)	0.338 (0.566)
*E*_*global*_	**13.052 (0.002)** ^**F<M**^	*3*.*652* (*0*.*068*)^**1st>2nd**^	0.308 (0.584)
*E*_*local*_	1.421 (0.247)	0.621 (0.438)	1.907 (0.180)

The statistical results were computed with a two-way linear mixed model with longitudinal time as within-subject fact, gender as between-subject factor and time by gender as interaction. The effect of age at 1^st^ scan, handedness, education level, and brain size were adjusted for all of these analyses. Post-hoc results of the global topological measures were detailed in Fig. 4. **Bold** indicates variables that are statistically significant (p < 0.05), *italic* indicates variables that show trend of significance (p < 0.10).

### Longitudinal gender difference on the regional properties

**Fig 5:**
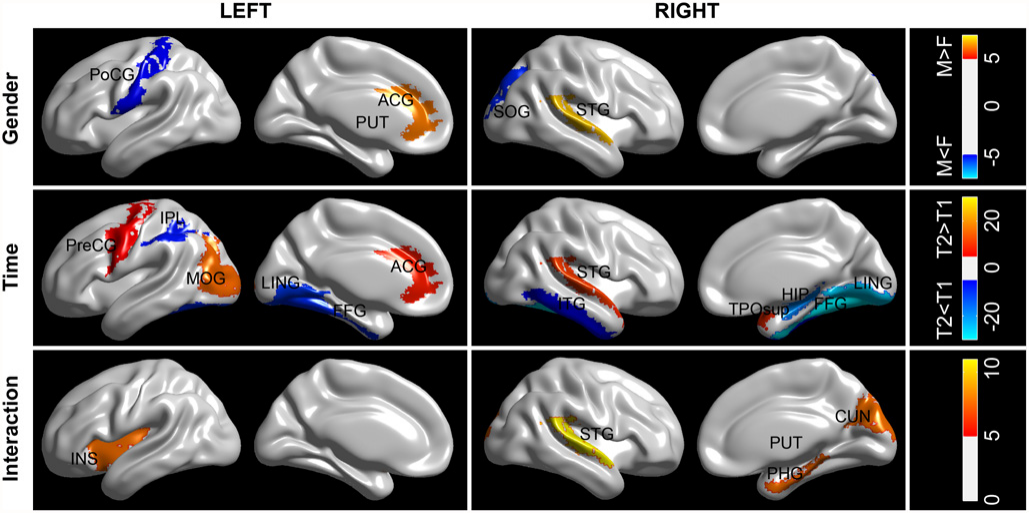
The spatial distributions of cortical regions showing significant effect of gender, longitudinal time, and gender-time interaction on the nodal betweenness centrality. The color represents F values. Significant regions are overlaid on inflated surface maps at the Medium view with BrainNet Viewer software (http://www.nitrc.org/projects/bnv). M = male, F = female. For the abbreviations of the cortical regions, see Table 1.

Two-way linear mixed model ANCOVA revealed a significant gender effect on the nodal characteristics of five regions (Fig. 5), whereby two nodes exhibited female advantage (the PoCG.L, and the SOG.R) and the remaining three presented male advantage (the left anterior cingulate gyri, [ACG.L], the PUT.L, and the right superior temporal gyrus, [STG.R]). Whilst few in number, these gender effects are in line with previous results in the baseline study. In addition, both positive and negative time effects were found on the nodal characteristics of selective brain regions across the cerebral cortex (12 in total). Among these altered brain regions, 7 regions (including the right hippocampus [HIP.R], the left inferior parietal lobe [IPL.L], the ITG.L and the bilateral LING and the bilateral FFG) showed decreased nodal betweenness between two scans, whereas 5 regions predominantly located in the left occipital and right temporal cortex (including the PreCG.L, the ACG.L, the MOG.L, the STG.R, and the temporal pole, superior part, [TPOsup.R]) revealed significantly increased nodal betweenness. Most of the identified regions (8 of 12) with a time effect resided in the association cortex, supporting the view that longitudinal changes are mainly characteristic of the association cortex as opposed to primary cortex [38]. Particularly, prominent reduction of nodal betweenness was revealed in our identified hub regions for both male and female subjects (i.e., the bilateral LING and the bilateral FFG). More interestingly, five regions exhibited significant gender-time interactions (including the left insula [INS.L], the right parahippocampal gyrus [PHG.R], the CUN.R, the PUT.R, and the STG.R). It is noted that most of these regions (4/5) resided in the right hemisphere; only one region exhibited significant gender-time interactions in the left hemisphere. The post-hoc analysis of the gender-time interaction revealed that this effect was attributed to a) different longitudinal trajectories between men and women, i.e., a significant increase of nodal efficiency of the INS was observed in females whereas a trend-wise decrease of nodal efficiency of the INS in males, or b) accelerated increase/decrease of nodal efficiency in one group compared to the counterpart, i.e., an accelerated decrease of nodal efficiency of the PUT.R in males.

## Discussion

In this study, we employed graph theoretical analysis of DTI data to investigate the gender effect on the structural brain networks in healthy adults and how it would be modulated over a longitudinal time interval. The main findings are as follows: 1) both males and females showed prominent small-world properties and shared several hub regions; 2) females demonstrated more economical small-world architecture, whereas males exhibited greater global efficiency; 3) a significant gender-time interaction was revealed in the weighted clustering coefficient; and 4) several specific brain regions exhibited different development trajectories between males and females. These findings are discussed in greater detail below.

### Small-world properties

Recent neuroimaging studies have suggested that human brain networks have small-world topologies (for a review, see [39]). The comparable and conserved small-world properties observed in this work further support these earlier studies and suggest that small-world topology is a fundamental principle for the anatomical organization of complex brain networks to maximize the power of information processing. Interestingly and in agreement with previous studies that have shown greater local clustering in cortical anatomical networks in females as compared with males [20,26,40], we also found a less economical small-world architecture in men than in women. Gur and colleagues found that females had a higher percentage of GM and a lower proportion of WM than males, which is independent of brain size [3,41]. They also found a stronger association between cognitive performance and WM volume in females, suggesting that females may make more efficient use of the available WM. Cerebral GM contains neuronal cell bodies, dendrites, and short protrusions which play an important role in regional information processing. The higher percentage of GM in women increases the proportion of brain tissue available for computational processes, which might contribute to the more optimal small-world architecture in females. In addition, we demonstrated a higher global efficiency in males, suggesting a predilection for global information integration. Using DTI, Ingalhalikar et al. demonstrated a male preponderance of intra-hemispheric connections bilaterally, whereas females only exhibited greater left-to-right frontal lobe connections [21]. Our observation of more efficient global information transfer in males might be attributed to the strengthened bilateral intra-hemispheric connections.

### Gender related differences in regional efficiency

Betweenness centrality is a network measurement that shows how “central” a node is to the network, based upon how many of the shortest paths go through the node. Nodes with high betweenness centrality may serve as way stations for network traffic or as centers of information integration [42]. Of note, most of the hubs identified in the present study were previously observed to have high regional efficiency or betweenness centrality in structural [26,28,43] and functional networks [44,45]. Males and females shared most of the identified hub regions which are predominately located in heteromodal and unimodal association cortices (Fig. 2). Association regions have proven to contribute to the integrity of multiple functional systems, such as attention and memory [37]. Our findings thereby provide further support for the notion that association cortices play a pivotal role in human structural brain networks. In addition, females showed higher betweenness centrality in four association and one of the primary cortices (the IFGtriang.L, the PoCG.L, the STG.L, and the bilateral SOG) whereas in males greater regional centrality was revealed in the SFGmed.L (Fig. 3). Using structural neuroimaging and volumetric analysis, Luders et al. found that females showed higher GM thickness in the left inferior frontal, bilateral parietal and superior occipital gyrus [46]. As mentioned above, a higher volume of GM corresponds to a larger proportion of brain tissue available for regional information processing. Our results of female advantage in these observed regions therefore represented more neural processing in female subjects. Interestingly, two well recognized language-related brain regions (the IFGtriang.L and the STG.L) showed higher nodal efficiency in females, which may underlie the advantages of female in verbal related functions [47,48]. Males were shown to have larger brain volume in frontomedial cortex [49] and putamen [50], which may explain the male predilection for nodal efficiency in the identified regions. Given the important role of the putamen in motor control, the predominant nodal centrality of putamen in males may contribute to the repeatedly reported male predilection in motor performance and tasks [51].

### Longitudinal gender effects on small-world properties

Consistent with the observations in the baseline study, similar gender main effects were also observed in the longitudinal study. Therefore, our results added to the earlier cross-sectional data [20,26] and provided further longitudinal evidence in revealing gender effects on structural brain networks. Furthermore, as expected, we showed a significant reduction of overall WM fibers in men and women across time. In accordance with our finding, convergent evidence suggests that WM decreases in a quadratic fashion with a greater rate beginning in adult midlife [52,53]. The alteration of WM fibers over time may impair the functional integration between distant brain regions, eventually triggering selective cognitive decline in late life. Therefore, our observation further supports the notion that cognitive deficit in aging arises from the disconnection of brain areas in addition to the selective aberrations of GM areas [54]. After factoring out the differences in overall WM fiber numbers, we found the topological organization of the brain networks of both males and females was adapted at the second scan point: the structural brain networks of both males and females retained the economical small-world properties. Somewhat contrary to our second hypothesis, the global network architecture failed to reveal significant longitudinal time effect. Only a trend-wise deficit of global integration was observed for both male and female subjects. This observation is in line with several recent studies on a large sample of healthy subjects aged from 18 to 80, where the brain network organization shifts to a more localized architecture in old age [43,55]. Taken together, although the structural brain networks for the middle-age adults preserved the optimal architecture, there was already some indication pointing to the development tendency toward a less economical topology with aging. Interestingly, a significant gender-time interaction was revealed in the weighted clustering coefficient, attributing to different development trends between males and females. Particularly, males showed a trend of improvement of their local clustering whereas females exhibited a significant decrease of local clustering, which was further validated in the FA-based structural network (S1 and S2 Tables). These findings thereby demonstrated that the longitudinal global organization of structural brain networks is modulated by gender in middle-age healthy adults. In contrast, in one recent cross-sectional structural brain connectivity study, only main effect of gender and aging was observed [26]. We speculate that the apparent inconsistencies might be due to the different designs of studies (longitudinal *vs*. cross-sectional). Further studies with longitudinal design therefore are needed to reconcile the apparent inconsistencies and confirm our findings.

### Longitudinal gender effects on the regional properties

The longitudinal sexual dimorphism of structural brain networks was further localized in terms of regional nodal betweenness centrality. We found that most of the cortical regions with significant gender effect were overlapped with the observations in the baseline dataset. In addition, we also showed that the ACG and the STG had higher nodal centrality in males compared to females. At baseline, a trend of gender effect on the nodal centrality of the ACG.L and the STG.R was revealed. The finding of greater nodal efficiency in the male the ACG was in accordance with previous observation of a greater nodal interconnectivity in the male paracingulate gyri [20]. Previous structural MRI studies showed that males had higher GM volume in the paracingulate gyri [56]. We speculated that it might be associated with the higher nodal centrality of the ACG in males as seen in our study. Using transcranial magnetic stimulation, Ellison and colleagues found the involvement of right superior temporal gyrus in serial visual search [57]. The higher nodal centrality of the STG.R might indicate a better performance of men in visual search related tasks. Essentially, the network organizational change results from the differential decline and relative preservation of specific regional anatomical connections in aging brain [26]. In this study, both negative and positive time effects on regional efficiency were found across cerebral cortex. Given that the global network architecture showed non-significant longitudinal time effect, these inhomogeneous developments may suggest a putative compensatory mechanism of cortical network reorganization over time. Similar results were also observed by several other groups [26,40,43]. Specifically, several brain regions (e.g., the IPL, the SOG and the STG) consistently exhibited an alteration of regional efficiency in this work and previous studies. Furthermore, we found that there were significant interactions between gender and longitudinal time on nodal efficiency of five regions (Fig. 5). Additional post hoc analysis of these regions with regard to significant interactions revealed that this effect was attributed to a) different development trajectories (e.g., the INS.L) or b) accelerated increase/decrease trajectories between males and females (e.g., the PUT.R). The findings are in accordance with one recent cross-sectional study on a large sample of 1460 subjects in which these cortical regions have been identified to exhibit significant age by gender interactions in GM volumes [58]. In one recent longitudinal study, Pfefferbaum et al. investigated the variations in developmental trajectories of regional brain volumes between healthy men and women, and found variations in the trajectory of the insula between males (cubic) and females (quadratic) as well as an accelerated linear decrease of brain volumes in putamen in males [15]. Therefore, our findings add to the literature about how local brain structures are modulated by gender over time, which might advance the understanding of the development of cognitive specificity in women and men.

### Methodological issues and further considerations

Several methodological issues need to be addressed. First, one main drawback is that the number of the participants who completed the longitudinal scan is relatively small. Although great consistency of the gender effect was observed between baseline and longitudinal results, future research with an independent larger study sample over a longer time frame is needed to confirm our observations. Second, the DTI deterministic tractography method was employed in this work to reconstruct structural brain networks. Although this method has been widely used, it has a limited capacity for resolving the fiber crossing issue and may result in a loss of the estimated fibers [59]. In one recent study, Buchanan et al. investigated the test-retest reliability of structural brain networks constructed from diffusion MRI and revealed that the probabilistic tractography outperformed the deterministic method in overcoming the fiber crossings and robustness to the image noise [60]. Therefore, further attempts could be conducted on structural brain networks reconstructed by probabilistic diffusion tractography methods [21,26]. Third, to control the influences of the total fiber number differences across subjects and investigate the salient topological differences between males and females beyond the simple gender-related differences in WM connectivity strength, a normalization approach was performed here prior to the network metrics estimation [28]. However, in weighted network analyses which incorporate the variations in the strength of connectivity into the network metrics estimation, significant gender effect in the WM microstructure might have some potentially pronounced effect on the network measures. It would therefore be important for future attempts to explore the progressive gender differences of structural brain networks with different weighting approaches [60]. Fourth, recent studies have suggested that the node definition by different parcellation scales might result in different properties of brain networks [61–63]. Therefore, graph analyses with different spatial resolution is encouraged in the future to provide more comprehensive information on the gender related topological differences of structural brain networks. Fifth, convergent evidence has revealed a close relationship between resting-state functional connectivity and the underlying structural connectivity (for a review, see [64]). It would be important in future studies to explore how the gender-related structural brain network differences are associated with the alteration of functional brain networks, and more importantly how they evolved over time, through simultaneously evaluating the topologies of functional and structural connectivity networks. Finally, as our study is one of the first exploratory investigations of progressive sexual dimorphism in brain network topology, we did not perform corrections for the multiple comparisons and focused on the interpretation of the general patterns of the findings. We also provided the exact statistical analysis results for the reader’s interpretation. In view of the modest size of the longitudinal samples used in this study, future research with a larger independent longitudinal sample is needed to confirm our observations.

In summary, in this longitudinal DTI study of sexual dimorphism in brain structures, we used graph theoretical analysis to investigate how the gender differences in topological organization of the structural brain networks in middle-age healthy adults were modulated over time. Our findings provide further brain connectome evidence to support the presence of sexual dimorphism in brain structures that may underlie gender differences in behavioral and cognitive functioning. Moreover, insights of the progressive gender differences in brain connectome may provide an important foundation to delineate the pathophysiological mechanisms underlying sex differences in neuropsychiatric disorders and to potentially guide the development of sex-specific treatments for these devastating brain disorders [4,65]. Future studies of structural brain networks in neuropsychiatric disorders must take into account gender differences in order to better appreciate the neural substrates underlying these potentially crippling conditions.

## Supporting Information

S1 Text Effects of different connectivity weight on network properties. (DOCX)

S1 Fig The small-world characteristics of the FN-based structural brain connectivity networks in male (blue) and female (red) subjects at baseline. Both groups showed prominent small-world properties, i.e., a much higher clustering coefficient and a similar characteristic path length compared to the matched random networks (Male-rand, Female-rand).
(TIF)

S1 Table Comparison of topological properties in the FA-weighted network at baseline. (DOCX)

S2 Table Comparison of the network metrics in the FA-weighted network with longitudinal scans between male (n = 13) and female (n = 15). (DOCX)
