# Supplementary material for: Progressive Gender Differences of Structural Brain Networks in Healthy Adults: A Longitudinal, Diffusion Tensor Imaging Study
Source: PLoS One. 2015 Mar 5;10(3):e0118857. doi: 10.1371/journal.pone.0118857 (PMC4350987; doi:10.1371/journal.pone.0118857)
Supplement: S1 Text — (DOCX) [file pone.0118857.s001.docx]

**S1 Text. Effects of different connectivity weight on network properties**

The fractional anisotropy (FA) value is an important index to evaluate the fiber integrity and exhibits a high correlation with conductivity. To evaluate the effects of connectivity metrics on the network properties, we also constructed the FA-weighted structural networks and performed the graph theoretical analysis on the global topological properties for each subject. We found that the results estimated from the FA-weighted networks were compatible with those of the FN-weighted networks reported in the main text (S1 and S2 Tables).

*Baseline*: Similar to the findings in the FN-weighted networks, significant male advantage in efficiency of global information integration was observed at baseline (*L_p_*, F_1, 65_ = 10.748, p = 0.002, and *E_global_*, F_1, 65_ = 7.198, p = 0.010). On the other hand, females showed significant higher small-world properties (*σ*, F_1, 65_ = 6.204, p = 0.015).

*Longitudinal*: Significant gender effect was observed in *L_p_*, (male < female, F_1, 22_ = 8.102, p = 0.009), *σ* (male < female, F_1, 22_ = 5.450, p = 0.029), and *E_global_* (male > female, F_1, 22_ = 6.598, p = 0.018). These results echoed with the findings in the cross-sectional data. For the longitudinal time effect, significant ongoing increase of characteristic path length (1^st^ < 2^nd^, F_1, 26_ = 5.839, p = 0.023) and decrease of *E_gloabl_* (1^st^ > 2^nd^, F_1, 26_ = 4.409, p = 0.046) were observed. A trend of significantly decreased of local efficiency (1^st^ > 2^nd^, F_1, 26_ = 3.763, p = 0.063) were also revealed. Interestingly and in agreement with the results of FN-weighted networks, a significant gender-by-time interaction was found in the weighted clustering coefficient (F_1, 26_ = 5.416, p = 0.028).

Taken together, these additional analyses provide convergent evidence for the validity of our findings reported in the main text.
