# Supplementary figures and images for: Progressive Gender Differences of Structural Brain Networks in Healthy Adults: A Longitudinal, Diffusion Tensor Imaging Study
Source: PLoS One. 2015 Mar 5;10(3):e0118857. doi: 10.1371/journal.pone.0118857 (PMC4350987; doi:10.1371/journal.pone.0118857)

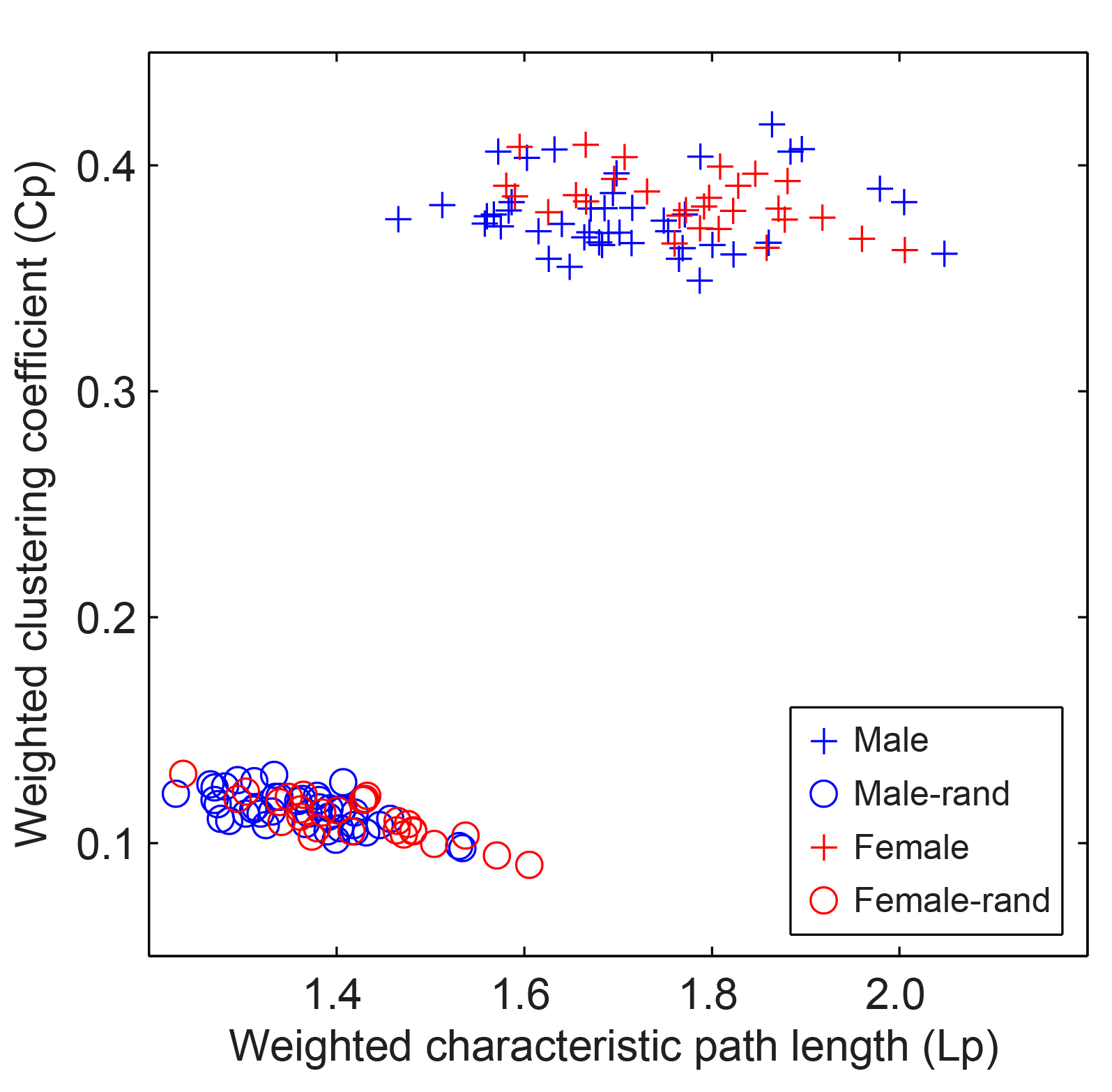

Supplement: S1 Fig — Both groups showed prominent small-world properties, i.e., a much higher clustering coefficient and a similar characteristic path length compared to the matched random networks (Male-rand, Female-rand). (TIF) [file pone.0118857.s002.tif]
