# Supplementary material for: Progressive Gender Differences of Structural Brain Networks in Healthy Adults: A Longitudinal, Diffusion Tensor Imaging Study
Source: PLoS One. 2015 Mar 5;10(3):e0118857. doi: 10.1371/journal.pone.0118857 (PMC4350987; doi:10.1371/journal.pone.0118857)
Supplement: S1 Table — (DOCX) [file pone.0118857.s003.docx]

**S1 Table. Comparison of topological properties in the FA-weighted network at baseline**

| Metrics | Male (n=43) | Female (n=28) | F_1,65_ (p-value) |
| --- | --- | --- | --- |
| *C_p_* | 0.205 ± 0.011 | 0.205 ± 0.007 | 0.309 (0.580) |
| *L_p_* | 4.348 ± 0.180 | 4.510 ± 0.124 | **10.748 (0.002)** |
| *σ* | 1.741 ± 0.108 | 1.822 ± 0.103 | **6.204 (0.015)** |
| *E_global_* | 0.255 ± 0.011 | 0.246 ± 0.012 | **7.198 (0.010)** |
| *E_local_* | 0.361 ± 0.012 | 0.358 ± 0.010 | 0.682 (0.412) |

The values of network metrics were shown with mean ± standard deviation. The statistical results were computed with a univariate analysis of covariance with age at scan, handedness, education level, and brain size as covariates. **Bold** indicates variables that are statistically significant (p < 0.05).
