# Supplementary material for: Progressive Gender Differences of Structural Brain Networks in Healthy Adults: A Longitudinal, Diffusion Tensor Imaging Study
Source: PLoS One. 2015 Mar 5;10(3):e0118857. doi: 10.1371/journal.pone.0118857 (PMC4350987; doi:10.1371/journal.pone.0118857)
Supplement: S2 Table — (DOCX) [file pone.0118857.s004.docx]

**S2 Table. Comparison of the network metrics in the FA-weighted network with longitudinal scans between male (n=13) and female (n=15)**

| Metrics | General linear mixed model factors | | |
| --- | --- | --- | --- |
|  | Gender | Time | Gender × Time |
|  | F_1, 22_ (p-value) | F_1, 26_ (p-value) | F_1, 26_ (p-value) |
| *C_p_* | 0.509 (0.483) | 0.164 (0.689) | **5.416 (0.028)** |
| *L_p_* | **8.102 (0.009) ^F>M^** | **5.839 (0.023) ^1st<2nd^** | 0.104 (0.749) |
| *σ* | **5.450 (0.029) ^F>M^** | 0.101 (0.753) | 0.203 (0.656) |
| *E_global_* | **6.598 (0.018) ^F<M^** | **4.409 (0.046) ^1st>2nd^** | 0.498 (0.487) |
| *E_local_* | 0.001 (0.989) | *3.763 (0.063)* **^1st>2nd^** | 0.428 (0.519) |

The statistical results were computed with a two-way linear mixed model with longitudinal time as within-subject fact, gender as between-subject factor and time by gender as interaction. The effect of age at 1^st^ scan, handedness, education level, and height were adjusted for all of these analyses. **Bold** indicates variables that are statistically significant (p < 0.05), *italic* indicates variables that show trend of significance (p < 0.10).
